# Dynamic release of gentamicin sulfate (GS) from alginate dialdehyde (AD)-crosslinked casein (CAS) films for antimicrobial applications

**DOI:** 10.1080/15685551.2016.1231037

**Published:** 2016-09-20

**Authors:** S. K. Bajpai, Farhan Ferooz Shah, M. Bajpai

**Affiliations:** ^a^ Polymer Research Laboratory, Department of Chemistry, Govt. Model Science College, Jabalpur, India

**Keywords:** Hydrogels, wound dressings, alginate dialdehyde, casein

## Abstract

In the present work, antibiotic drug gentamicin sulfate (GS) has been loaded into alginate dialdehyde-crosslinked casein (CAS) films for wound dressing applications. The films have been characterized by Fourier transform infrared spectroscopy, X-ray diffraction analysis and scanning electron microscopy. The dynamic release of model drug GS has been investigated in the physiological fluid at 37 °C. The drug release data has been interpreted in the terms of various kinetic models such as Power function model, first order model and Schott model. The release data was found to be well fitted by Schott model. The various diffusion coefficients are also evaluated. The adsorption of model therapeutic protein BSA on the film has been investigated. The maximum adsorption is found to be 5.7 mg/cm^2^.The films were tested for their antibacterial and anti-fungal action. Finally, the *in vivo* wound healing study was carried out on Albino wistar rats.

## 1. Introduction

Casein is a milk protein, comprising of around 94% protein and 6% low molecular weight compounds collectively known as colloidal calcium phosphate.[1] Casein possesses a number of favorable properties that make it a suitable candidate for biomedical applications.[2–7] These properties include hydrophilicity, biocompatibility, lack of toxicity, and presence of various functional groups susceptible to chemical modifications.[8] The water insoluble casein-based films are usually produced by crosslinking of casein with aldehyde groups containing cross-linkers such as glutaraldehyde and glyceraldehyde [9] or with enzymes like transglutaminase or tyrosinase.[10] However, as per reports, toxicity is a major issue using aldehyde cross-linkers.[11] In addition use of enzymes as cross-linkers is also very expensive. Therefore use of some non-toxic cross-linker is required to prepare casein based hydrogels intended to be used in biomedical and food applications.

In our previous work,[12] we prepared alginate dialdehyde (AD) by periodate oxidation of sodium alginate (SA) under controlled conditions and the dialdehyde, thus produced, was used as a crosslinking agent to fabricate casein films. Alginate is a biopolymer and has fair reputation as a non-toxic and biocompatible material.[13] It has been widely employed as wound dressing material for the delivery of different antimicrobial ingredients.[14,15] Similarly, its oxidized product, i.e. AD, has also been employed as a cross-linker to prepare protein-based hydrogels.[16,17] In continuation to our previous work, we hereby report detailed investigation of gentamicin sulfate (GS) release from the AD-crosslinked-casein hydrogels for antimicrobial applications. The dynamic drug rerlease data has been analyzed using various kinetic models. The kinetic drug release data, obtained from the release of entrapped bioactive ingredient from the polymeric films, need to be interpreted in the terms of various kinetic models developed. The useful information, extracted from the various parameters associated with a model, helps to obtain the films with maximum therapeutic efficacy. In a study by Maji et al. [18], matrix type transdermal patches were prepared using alprazolam as a model drug and employing the combinations of chitosan-polyvinyl alcohol (CS-PVA) cross linked with Maleic anhydride. The *in vitro drug* permeation followed Higuchi kinetics as its coefficient of correlation value predominated over zero order and first order kinetics. Also the diffusion coefficient of release profiles had a value of nearly 0.5, which indicated Fickian transport diffusion. In another study,[19] the dynamic release of Lidocaine Hydrochloride from poly(L-lactide) films was modeled using semi-empirical Higuchi, Korsmeyer–Peppas, and Gallagher–Corrigan models. The regression values for all the three models were fairly high, thus indicating suitability of these models. Most recently, Gustafson et al. [20] has reported controlled release of vancomycin from oligo(poly(ethylene glycol)fumarate)/sodium methacrylate charged copolymeric film and analyzed the drug release data using a phenomenological mathematical model based on a stretched exponential function. The cumulative release *F*(*t*) is given by *F*(*t*) = *C*[1 −exp(−(*t*/*τ*)*b*], according to that model, where *C* is the maximally achieved cumulative release (100%) and *τ* is the time when (1-e-1) *C* = 0.632*C* is achieved. In another study,[21] dynamic release of gentamicin from chitosan films was investigated under physiological conditions and the data was analyzed using Power functional model. Based on the values of release exponent ‘n’, a correlation was established between the mechanism of drug transport and fraction of drug bound to the polymeric chains within the film matrix.

**Figure 1.**
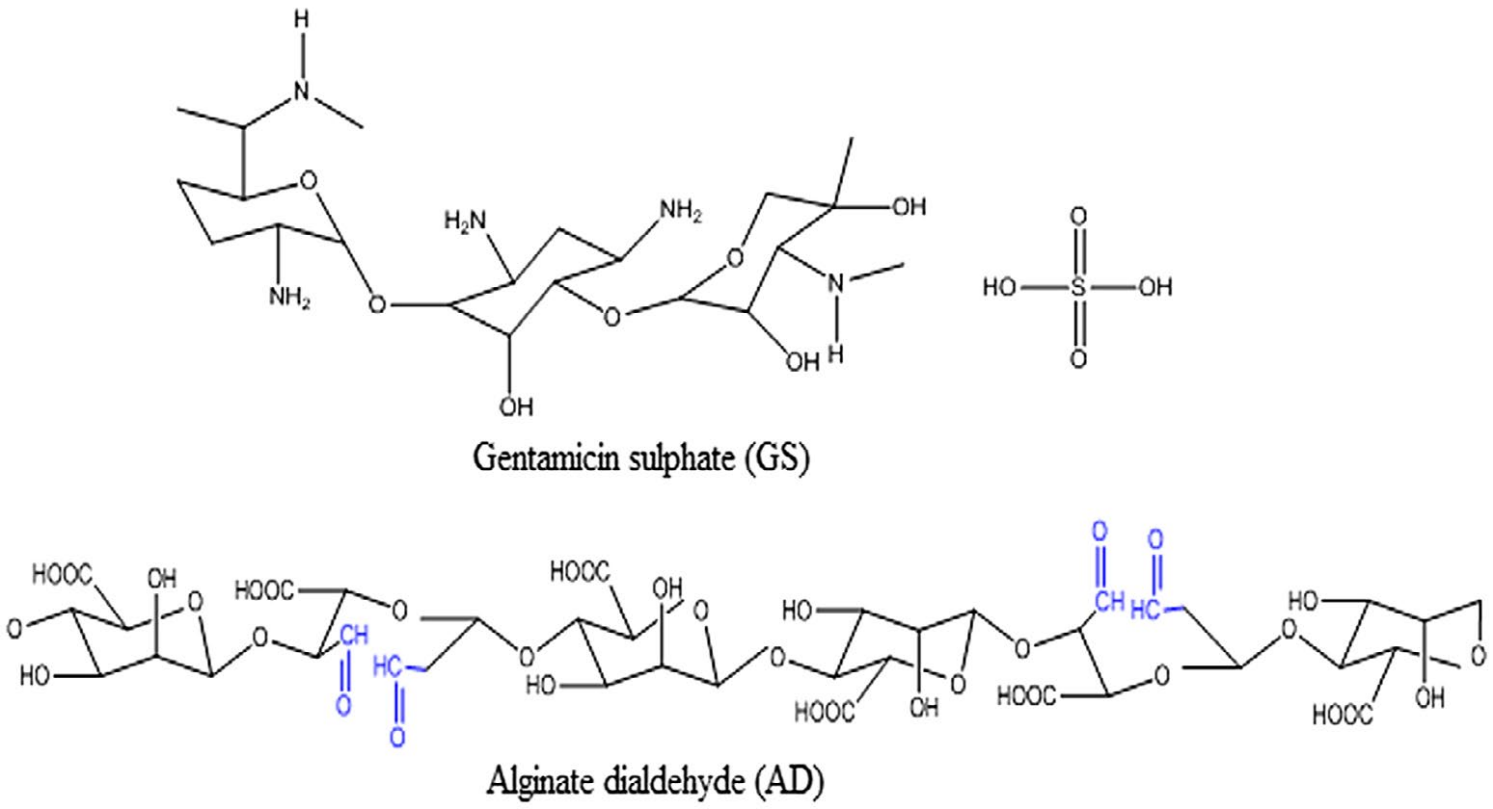
The structures of drug GS and the AD.

Gentamicin is an aminoglycoside antibiotic complex produced by fermentation of *Micromonospora purpurea* or *M. echinospora*. It is a mixture of three major components designated as C1, C1a, and C2.[22] Gentamicin is used as the sulfate salt. Each component consists of five basic nitrogen and requires five equivalents of sulfuric acid per mole of gentamicin base. The structures of drug GS and the AD are shown in Figure 1.

## 2. Materials and method

### 2.1. Materials

SA (molar mass of 150,000 Da and M/G ratio of 1.62, Hi Media Chemicals, Mumbai, India), casein powder (Cas; alkali soluble, with purity of 99.6%, Research Lab, Pune, India), potassium metaiodate (KIO_3_;analytical grade, Hi Media, Mumbai, India), ethylene glycol (Merck, Mumbai, India) were obtained in excellent conditions and used as such. Other chemicals such as sodium hydroxide, sodium dihydrogen phosphate, were obtained from Central Drug House; Mumbai, India. The antibiotic drug GS was purchased from local medical shop (Batch No.: FGLI-089). The double distilled water was used throughout the investigations.

### 2.2. Methods

#### 2.2.1. Preparation of AD

The alginate was oxidized using potassium iodate using the method described elsewhere.[23] In a typical experiment, 3 g of SA was dissolved in 100 mL of pre-warmed distilled water under mild stirring for a period of 2 h to ensure its complete dissolution. Now, 3.5 g of KIO_3_ was added under gentle stirring and the reaction mixture was placed in dark for a period of 12 h. Thereafter, the mixture was taken out and the reaction was quenched by the addition of 2.5 mL of ethylene glycol. The oxidized alginate was purified by precipitation with the addition of NaCl (2.5 g) and 100 mL of ethanol. The polymer was again dissolved in water (100 mL) and re-precipitated by the addition of ethanol (200 mL) in the presence of NaCl (1.00 g). The process was repeated using NaCl (0.50 g) and the polymer was precipitated with acetone (100 mL) under its sodium salt form. Finally, the precipitate was washed in ethanol (100 mL) under stirring during 15 min, isolated and dried at room temperature under vacuum.

#### 2.2.2. Preparation of AD-crosslinked casein films

In brief, 0.75 g of casein powder was dissolved in 15 mL of 1.5% NaOH solution and to this definite quantity of cross-linker AD was added under mild stirring for 15 min to ensure complete dissolution. The reaction solution was poured into Petri plate and allowed to keep in electric oven (Tempstar, India) for a period of 6 h at 60 °C. The film, thus formed, was peeled off and kept in a dust-free chamber for further use. In all, three films with varying amounts of cross-linker AD were prepared. The films were designated as (AD-X-CAS)_40_, (AD-X-CAS)_64_, and (AD-X-CAS)_76_, respectively, where the number out of the parentheses, denotes the percent cross-linking in the film.

The percent cross-linking was determined by the ninhydrine assay. In brief, pieces of an uncross-linked casein film and above three crosslinked films were placed in 20 mL of 1% ninhydrine solutions and allowed to heat at 60 °C for a period of 1 h. The final volume of each solutions was made to 50 mL with distilled water and the absorbance was determined spectrophotometrically (Genysis, USA) at 570 nm. The % free amino groups (FAG) were determined using the following expression:

(1) $$\%\text{FAG}=\frac{\text{Absorbance of solution containing crosslinked film}}{\text{Absorbance of solution containing casein film}}\times 100$$

The percent cross-linking of each film was expressed as (100-FAG).

#### 2.2.3. Preparation of GS loaded films

The antibiotic drug GS loaded films were prepared by the method of equilibration, which is supposed to be safer than the method which involves addition of the drug into the polymerization mixture. In a typical experiment, a pre-weighed piece of film sample (AD-X-CAS)_64_ was placed in drug solution of known concentration and equilibrated for a period of 24 h. Thereafter, the film was taken out and the volume of the drug solution was adjusted to the initial value by addition of distilled water. The equilibrium concentration of the drug solution was determined by measuring its absorbance and comparing it with the absorbance of standard solution. Finally, the amount of drug loaded in to the film was expressed as micro mol per g of film. The drug loaded films were designated as (DA-X-CAS)_475_, (DA-X-CAS)_1190_, and (DA-X-CAS)_2200_ respectively, where the number in sub-script denotes the micro mol of drug present in 1 g of polymer film.

### 2.3. Characterization of films

The Fourier transform infrared (FTIR) spectra were recorded with an FTIR spectrophotometer (Shimadzu, 8400, Japan) using KBr. The surface morphology of the plain and drug-loaded films was determined by scanning electron microscopy (SEM). The X-ray diffraction (XRD) method was used to measure the crystalline nature of films. These measurements were carried out on a Rikagu Diffractometer (Cu radiation = 0.1546 nm) running at 40 kV and 40 mA. The diffractogram was recorded in the range of 2 from 3 to 500 at the speed rate of 2°/min.

### 2.4. Drug release studies

The pre-weighed piece of drug-loaded film was placed in 25 mL of release medium (i.e. physiological fluid) at 37 °C. After regular time-intervals, film was transferred into fresh release medium, and the amount of drug released was determined spectrophotometrically at 254 nm. The quantity of drug was calculated using Lambert–Beer’s plot obtained for drug solutions of known concentrations. The ratio of volume of release medium to mass of film was maintained at a constant value of 250 (mL/g).

### 2.5. Antimicrobial study

The biocidal activity of GS loaded film was investigated by the method of ‘zone of inhibition’ as described elsewhere.[24] In brief, appropriate number of colony-forming units (CFU) of microbes (5 × 109 CFU/mL of *E. coli*) were cultured on a nutrient agar plate supplemented with a circular piece of test film i.e. (AD-X-CAS)_1190_ at the center of the plate. The plate was examined for a possible zone of inhibition after incubation at 37 °C for a period of 24 h. The plate, supplemented with plain film was considered as control.

We also carried out the antifungal activity of the film sample (AD-X-CAS)_1190_ against *Candida albicans*, and *Candida parapsilosis*. A 14-day-old culture was obtained from Fungal Disease Diagnostic Center, Jabalpur (India). For disc diffusion test, films were cut into disc shape with a diameter of 5 mm, then sterilized by autoclaving for 30 min at 120 °C, and finally placed on different cultured agar plates. The plates were incubated for 1 day at 37 °C in an incubation chamber.

### 2.6. Oxygen permeability

Oxygen penetration through films was performed by placing each film on top of open 250 mL-flasks (test area: 1.075 × 10^−3^ m^2^) containing de-ionized water. The negative and positive controls were the closed flask with an airtight cap and the open flask, respectively. The flasks were placed in an open environment under constant agitation for 24  h. Dissolved oxygen in water samples were analyzed according to Winkler’s method. Oxygen permeability (OP) (g/m^2^ day) was expressed as the amount of oxygen penetration through the film during 24 h.[25]

### 2.7. Bovine serum albumin adsorption

Adsorption of BSA onto AD-X-CAS film was investigated using batch model experiment. A series of aqueous solutions of BSA of known concentrations, ranging from 0.5 to 8 mg/mL were prepared. Now, a pre-weighed piece of film was placed in 25 mL of protein solution and kept in incubator (Temp star, India) for a period of 24 h to attain the equilibrium. Finally, the film was taken out, and the initial and the equilibrium concentrations of BSA were determined spectrophotometrically at 280 nm (Genesys, USA). The amount of BSA adsorbed per g of film (*q*_e_) was determined using the following expression:

(2) $$q_{\text{e}}=\frac{(C_{0}-C_{\text{e}})V}{m}\frac{\text{mg}}{\text{g}}$$

where *C*_0_ = Initial concentration of BSA solution (mg/L); *C*_e_ = Equilibrium concentration of BSA solution (mg/L); *V* = Volume of solution taken for adsorption (L); *M* = Mass of the film (g).

### 2.8. In vivo wound healing study

Albino wistar rats of either sex were used in the studies. Animals were housed under standard conditions of temperature, (25 ± 2°) and light, (approximately12/12 h light-dark cycle), fed on standard diet and given water ad libitum. Animal study protocols were approved by Institutional Animal Ethics Committee, Shri Ram Institute of Technology-Pharmacy, Jabalpur.

Animals were divided into two groups comprising of six animals in each as:

Group I: animals treated with plain film.

Group II: animals treated with (AD-X-CAS)_1190_ patch.

Excision wound was inflicted immediately on the rats under light chloroform anesthesia. Full skin thickness was excised with the help of surgical blade from the back of the central trunk marked area in order to get a wound measuring about 4 cm^2^. After achieving complete hemostasis by blotting the wound with cotton swab soaked in warm saline, the animals were placed singly in individual cages. Animals were treated once with drugs/formulations as stated above. The wound was observed daily until complete wound enclosure occurs.

### 2.9. Histological evaluation of healed wounds

The skin specimens from wounds healed areas were fixed in 10% buffered formalin and processed by paraffin tissue processing machine. The healed skin was under microscope by taking a 5 μm section followed by staining with hematoxylin and eosin.

## 3. Results and discussion

### 3.1. Preparation of AD-crosslinked casein films

**Figure 2.**
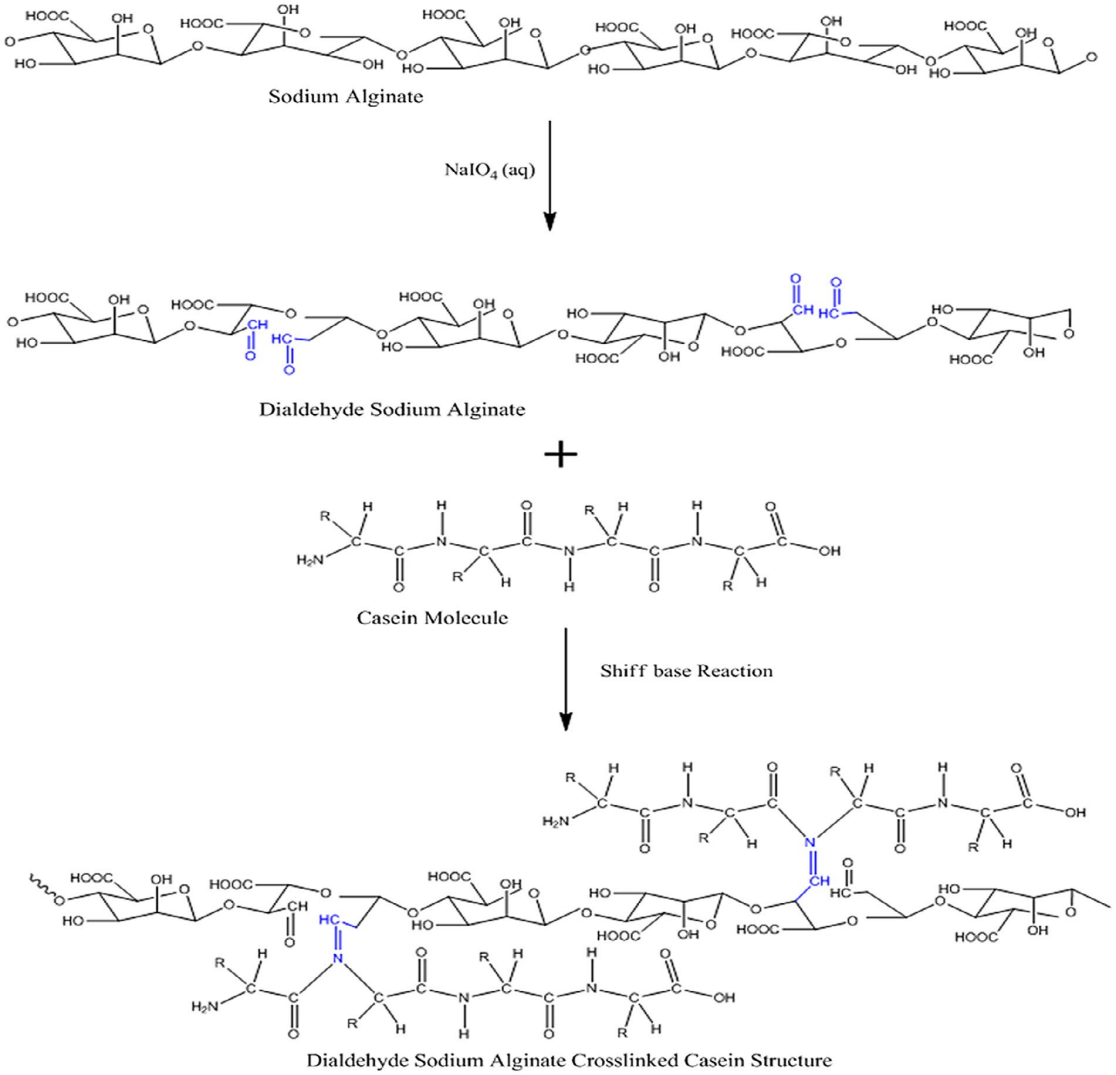
Scheme, showing the overall formation of the film.

The AD-crosslinked-Cas films were prepared by the Schiff base formation reaction between the aldehyde group of OA and –NH_2_ groups of casein. The method was similar to that reported by Saraswathy et al. [26]. A detailed protocol for the preparation has already been discussed in our previous work. However, a scheme, showing the overall formation of the film is shown in Figure 2.

### 3.2. Preparation of drug-loaded films

**Figure 3.**
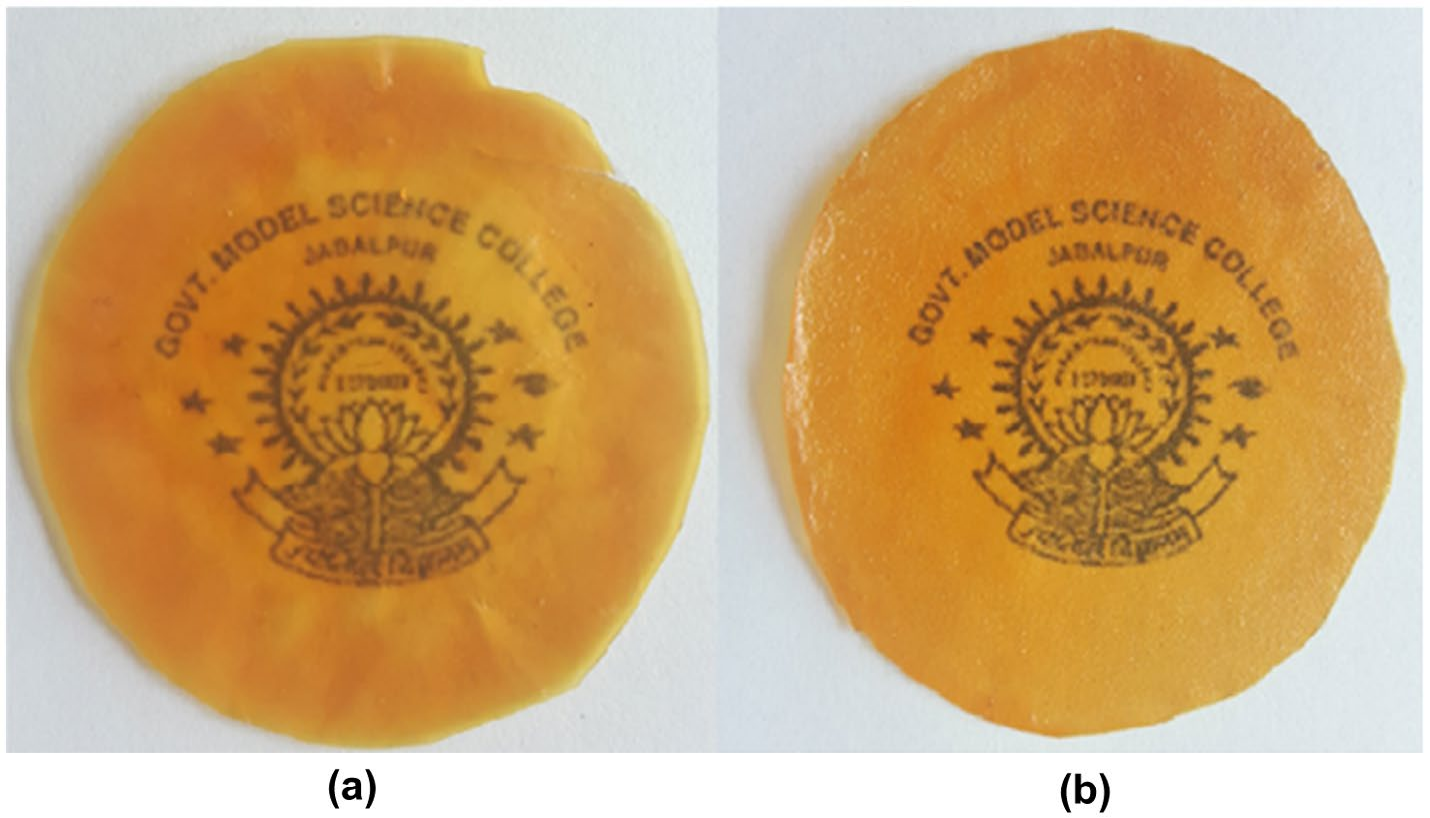
The optical images of (a) plain and (b) drug loaded films.

In this work, plain (AD-X-CAS) films have been loaded with drug GS by the method of equilibration. The method involves, preparation of plain films, leaching out of unreacted impurities by equilibration in distilled water for a period of 72 h, followed by their immersion in drug solution of known concentrations for entrapment of drug and then their drying till constant weight. The optical images of plain and drug loaded films are shown in Figure 3(a) and (b), respectively. It can be seen that the plain as well as drug loaded films are transparent to a satisfactory extent.

### 3.3. Characterization of films

**Figure 4.**
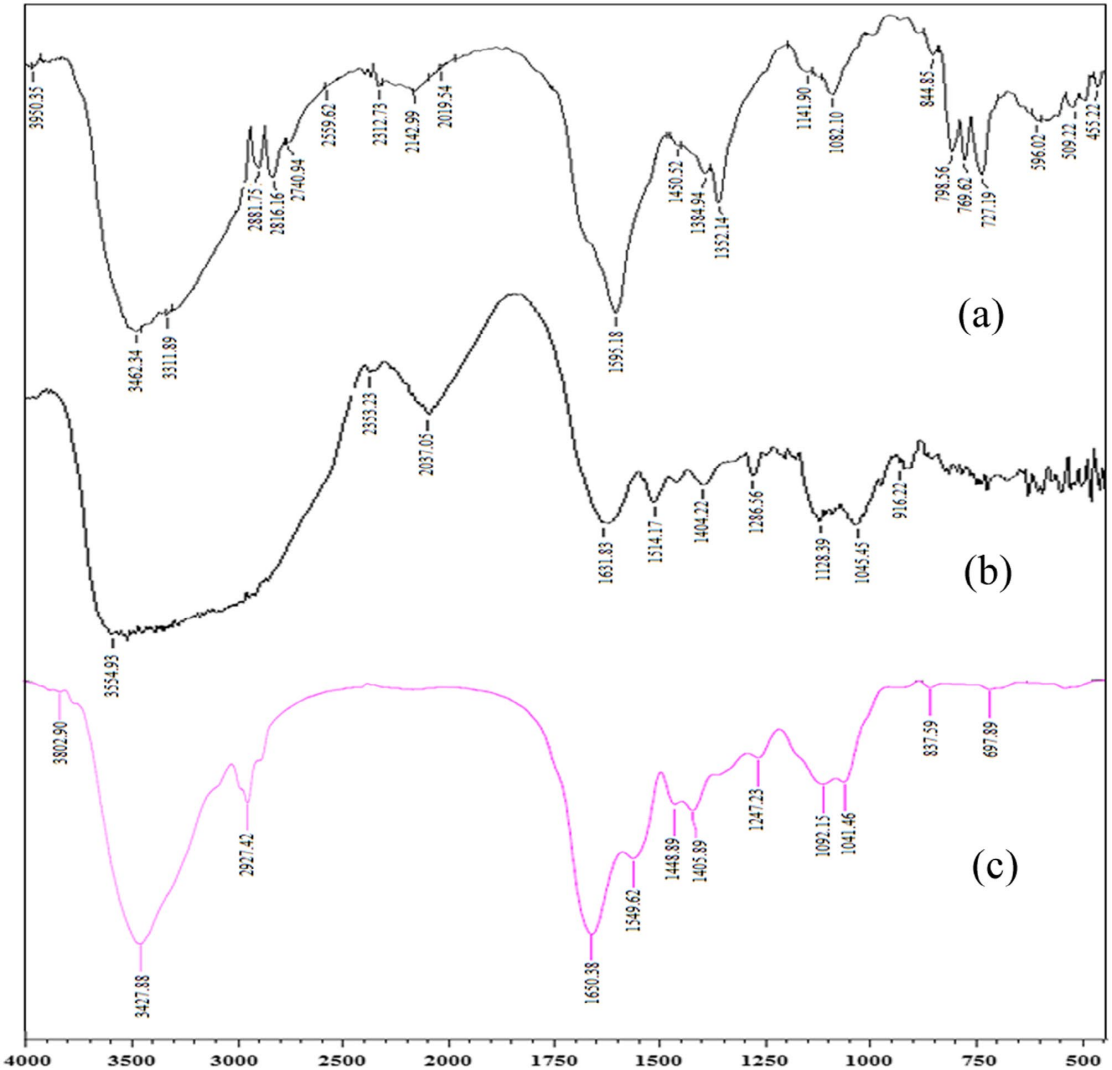
FTIR spectrum of (a) AD-cross-linked casein film (b) GS, (c) GS loaded AD-cross-linked casein film.

From the FT-IR analysis of the target compounds, Figure 4(a) and (c) characteristic vibrations of >C=N– at 1549–1450 (cm^−1^), respectively. The hydroxyl stretching frequency in all the spectra are indicated by the vibration at 3827–3200 cm^−1^. The appearance of characteristic peak of –NH_2_ functionality is obvious by looking at the vibrational wave numbers 3802–3462 cm^−1^. The carbonyl functionality is gestured by the frequency range of 1650–1595. In Figure 4(b) the main characteristic SO_2_ grouping is showed by the *v*_as_(SO_2_) at 1404 cm^−1^ and *v*_s_(SO_2_) 1296 cm^−1^ in the target drug and is seemable in the final loaded compound as well (Figure 4(c)), apperaing at 1448 and 1405 cm^−1^. Hence, the overall IR studies support the success of GS load in AD-crosslinked casein film.

**Figure 5.**
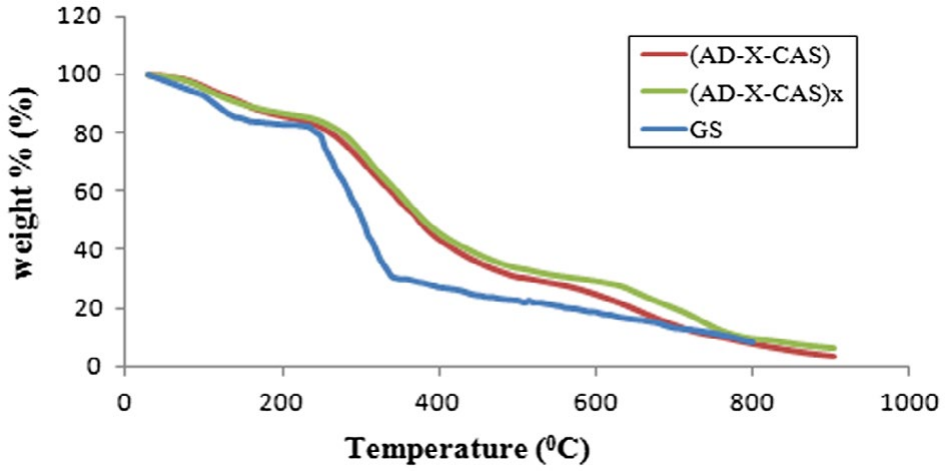
Thermal stability profiles for plain sample (AD-X-CAS), drug-loaded sample (AD-X-CAS)x and pure drug GS.

The results of thermo gravimetric analysis are shown in Figure 5. It can be seen that the thermo-grams of plain sample (AD-X-CAS) and drug-loaded sample (AD-X-CAS)x almost coincide, thus indicating that there is not any change in the thermal stability of the films. This may be attributed to the fact that thermal degradation of drug GS also occurs to almost same extent within the temperature range studied. For comparison, TGA of pure drug GS is also given. It can be seen that addition of GS in the plain film does not cause any noticeable different in the thermal stability of the resulting film. This is attributable to the fact that drug possesses almost same degradation profile.

**Figure 6.**
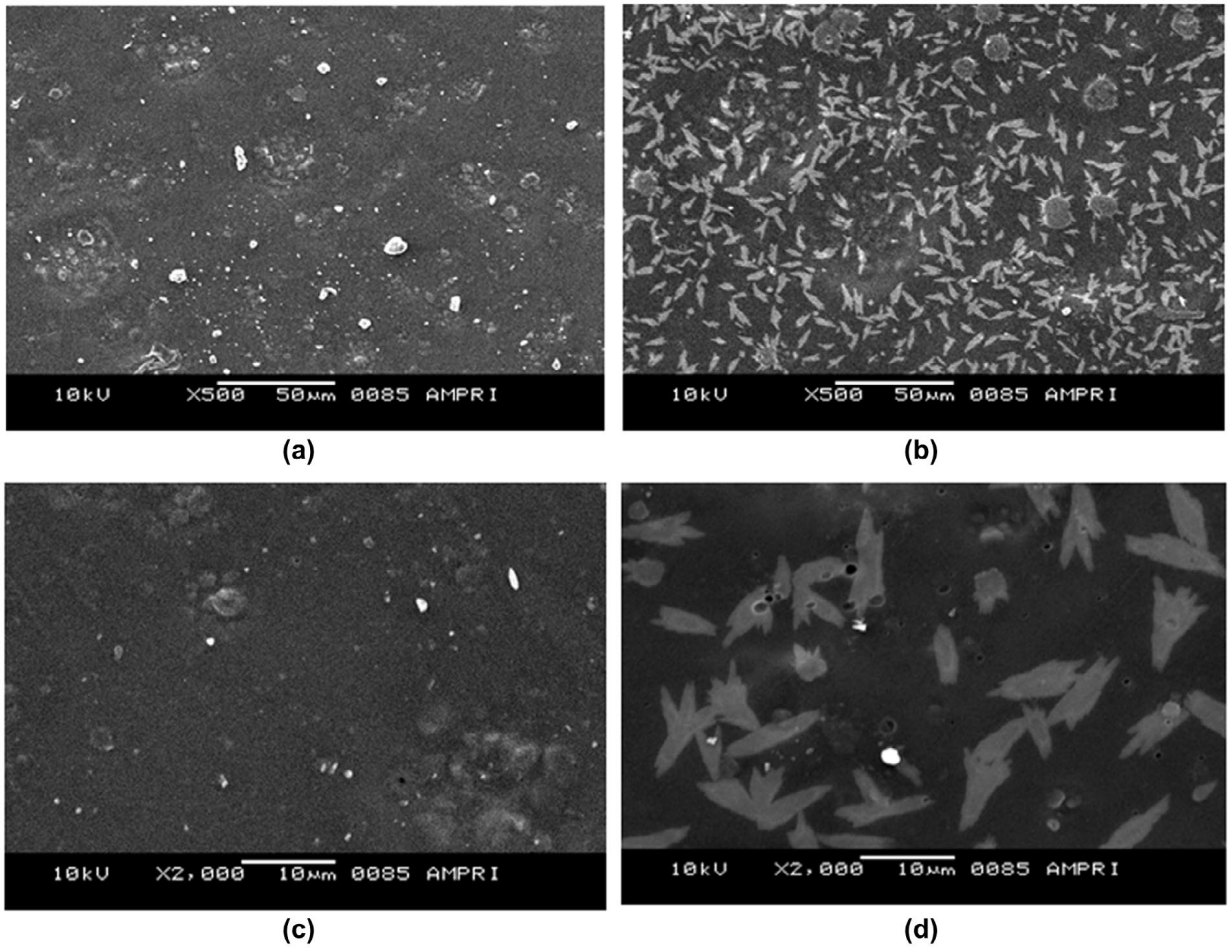
SEM images of plain (AD-X-CAS) film and GS loaded (AD-X-CAS) film at (a) and (b) 500×; (c) and (d) 1000× magnifications respectively.

SEM was performed to see any possible change in the surface of the films after drug loading. The SEM images of plain and drug loaded films are shown in Figure 6.

**Figure 7.**
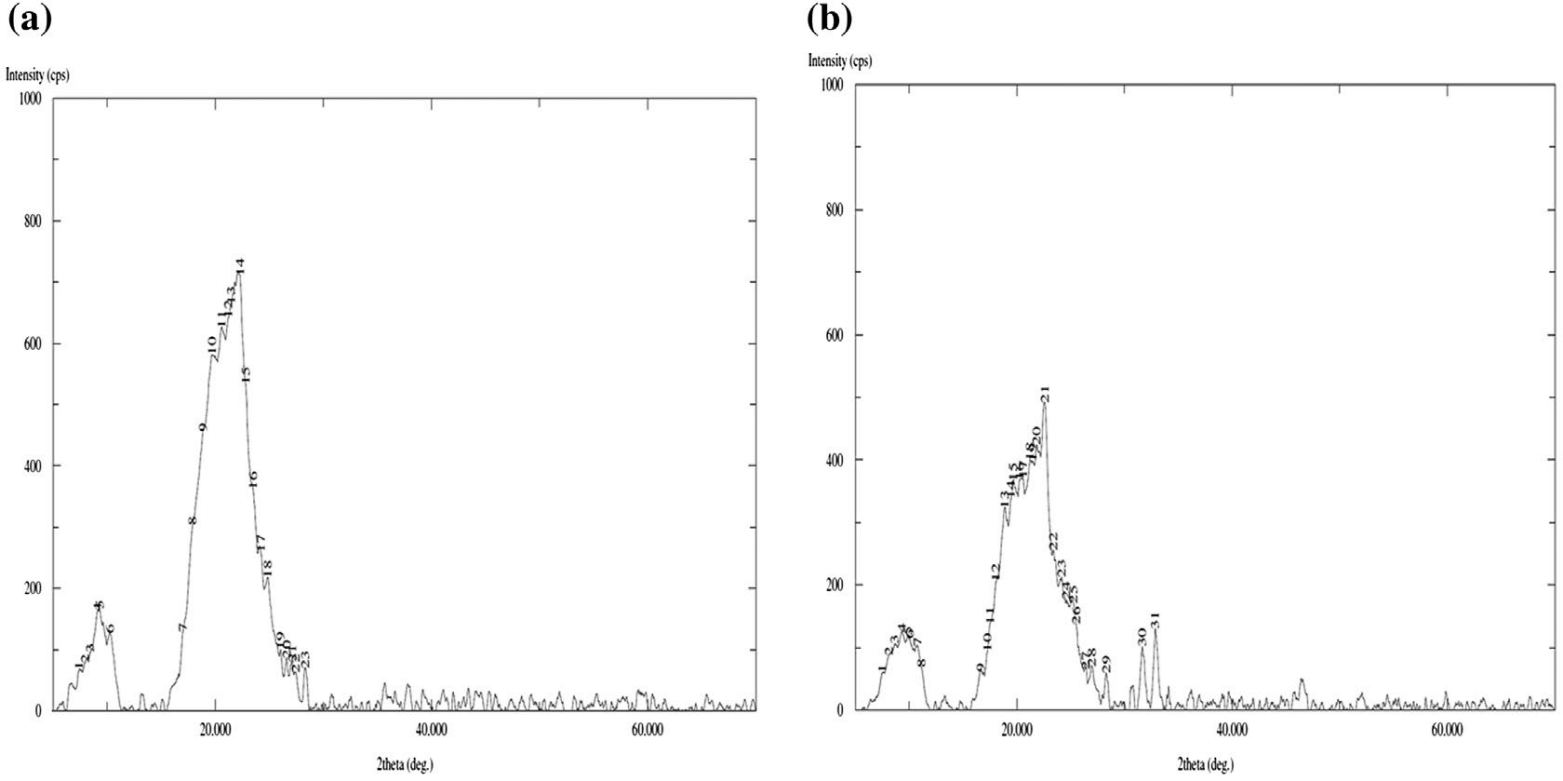
XRD patterns of (a) plain (AD-X-CAS) and (b) drug loaded film samples.

The images, shown in Figure 6(a) and (b) are that of plain and GS loaded films, with 500× magnifications over a bare scale of 50 μm. It can be seen that the surface of the plain film is almost smooth with appearance of some granules throughout, which might be due to presence of some agglomerations of casein particles. The image (b) of drug-loaded film with same degree of magnifications shows drug particles distributed throughout the film matrix. The images (c) and (d), obtained with 2000× magnifications and with bar scale of 10 μm also support the same observations. In the plain film some agglomerations can be seen while in the drug-loaded film irregular shaped drug particles can be seen. It is also noteworthy that both of the films do not show presence of any cracks or uneven surface texture. Finally, the XRD patterns of plain (AD-X-CAS) and drug loaded film samples are shown in Figure 7(a) and (b) respectively. The scattered broad pattern, observed in Figure 7(a), is indicative of the amorphous nature of casein as has also been reported elsewhere.[27] Furthermore, The XRD pattern of drug loaded film also shows diffused pattern with no sharp peaks, thus indicating amorphous nature.

### 3.4. Drug release studies

**Figure 8.**
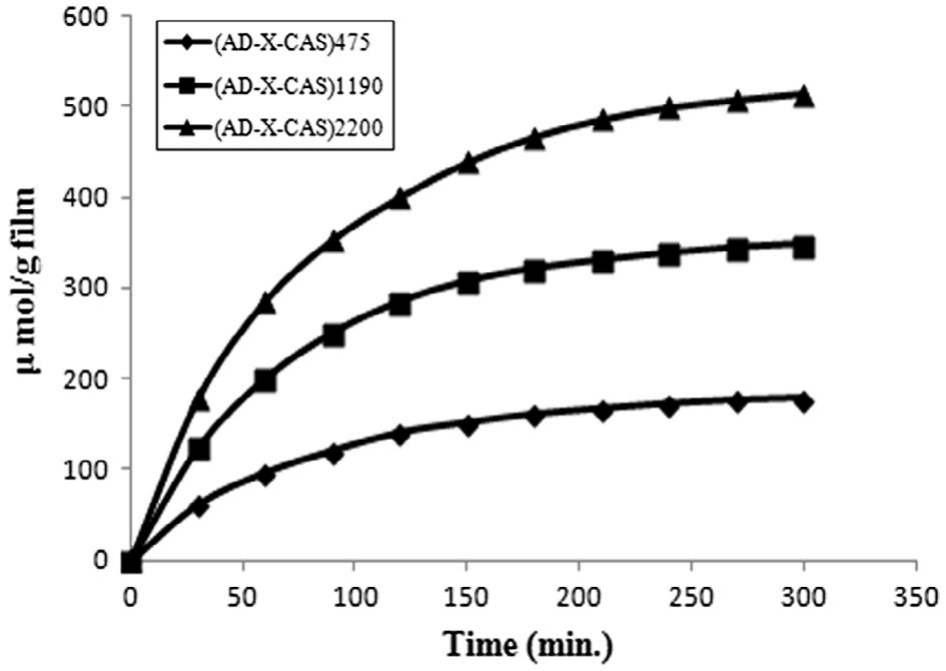
Dynamic release of drug GS from the samples (AD-X-CAS)_475_, (AD-X-CAS)_1190_ and (AD-X-CAS)_2200_ in the PBS of pH 7.4.

The dynamic release of drug GS from the samples (AD-X-CAS)_475_, (AD-X-CAS)_1190_ and (AD-X-CAS)_2200_ in the PBS of pH 7.4 is shown in Figure 8. It can be seen that as the drug content in the film increases, the amount released also increases. This is simply attributable to the fact that high drug content in the film produces a higher concentration gradient across the interface, and therefore a faster release is observed. The total amount of drug released from the samples was 271.54, 432.769, and 584.739 μmol/g respectively.

In order to determine the mechanism of drug transport from the films, the well-known ‘Power function model’ was applied,[28] according to which:

(3) $$\frac{M_{t}}{M_{\infty }}=k\;t^{n}$$

where *M*_*t*_ is the amount of drug released at time *t* and *M*_∞_ is the total release; *n* and *k* are release exponent and gel characteristic constant respectively. The logarithmic form of Equation (2) is:

(4) $$\ln F\left(M_{t}/M_{\infty }\right)=\ln k+n\ln t$$

**Figure 9.**
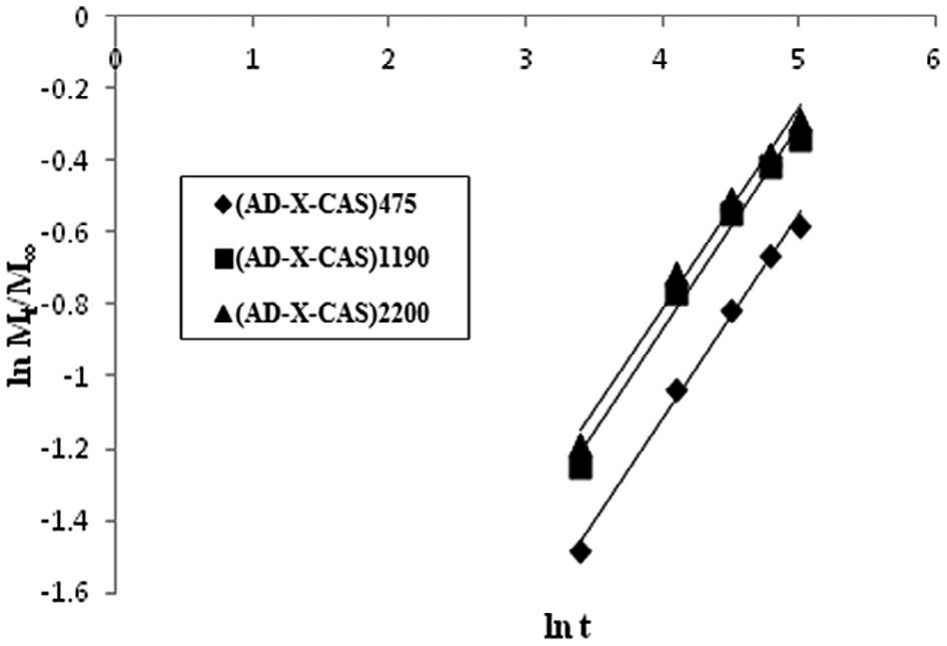
Interpretation of dynamic drug (GS) release data by Power function law.

The linear plots, obtained between ln *t* and ln *M*_*t*_/*M*_∞_, are shown in Figure 9.

The related parameters, *n* and *k*, obtained using slope and intercepts, are given in Table 1 along with the regression values.

**Table 1.** Parameters of Power function model.

Sample	*n*	*k* × 10^2^	*R*^2^
(AD-X-CAS)_475_	0.453	5.420	0.960
(AD-X-CAS)_1190_	0.432	7.584	0.937
(AD-X-CAS)_2200_	0.447	7.502	0.957

**Figure 10.**
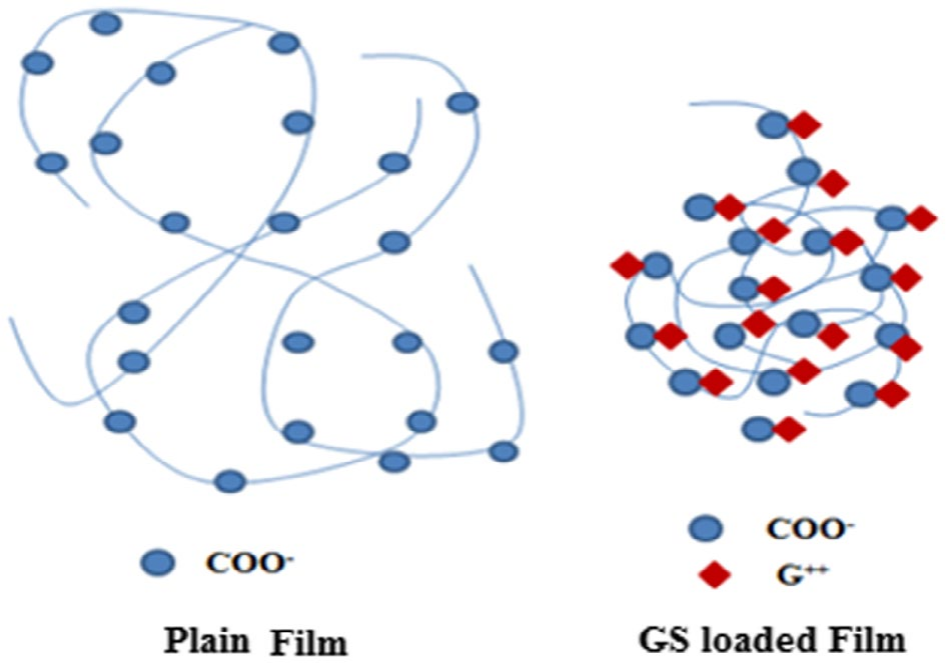
Effect of presence of cationic drug GS on the chain relaxation of casein chains.

A close look at the values displayed reveals that the release exponent ‘n’ lies between 0.43 and 0.45, thus indicating a Fickian transport mechanism. This suggests that drug GS is released from the film via a totally diffusion controlled manner. These results are quite unsupported from the water absorption data of these films, as reported earlier.[12] The equilibrium swelling ratio (SR) of the plain (AD-X-CAS) film was found to be 26.15 ± 1.69 g/g with a swelling exponent ‘n’ of 0.72, thus indicating a non-Fickian or chain relaxation controlled swelling behavior. We also measured the SR of the drug-loaded sample during drug release study and found that the drug loaded film sample demonstrated very little SR as compared to the plain (i.e. without drug) film sample of the same composition and with the same degree of cross-linking. The three samples did not swell appreciably as compared to blank samples of same composition and cross-linker concentration. The SRs of plain and three drug loaded samples were found to be 10.23, 1.10, 0.95, and 0.88 g/g respectively. This can be attributed to the strong intermolecular complexation due to hydrogen bonding between the carboxylic groups of casein molecules and the highly polar cationic drug. Moreover, these strong intermolecular forces of attraction screen the mutual repulsive forces among negatively charged –COO^−^ groups along the casein chains, thus weakening the chain relaxation process, which was dominant during the course of swelling of plain hydrogel films. This ultimately discourages the entrance of incoming water molecules, thus resulting in low degree of swelling as well as drug release. Almost similar trend was also reported by Thakur et al. [29], who studied release of GS from poly(acrylamide-co-acrylic acid) hydrogels .The effect of presence of cationic drug GS on the chain relaxation of casein chains is well illustrated in Figure 10.Thus, it can be concluded that presence of GS within the film matrix not only lowers the SR but it also makes the release process as Fickian-controlled.

Realizing that Power model is only applicable for the initial 60% release data, we also applied Schott kinetic model on the release data. According to this model, release rate at any time is directly proportional to the quadratic of the amount of drug released before the attainment of equilibrium state [30]:

(5) $$\text{d}M_{t}/\text{d}t=k_{2}{\left(M_{\infty }-M_{t}\right)}^{2}$$

where ‘k_2_’ is the second order drug release rate constant. Integration of above equation yields:

$$t/M_{t}=1/k_{2}M_{\infty }^{2}+t/M_{\infty }$$

Or

(6) $$t/M_{t}=A+B\;t$$

where *A* and *B* are coefficients with following significance: at a long retention time *B*. *t* >> *A* and therefore *B* = 1/*M*_∞_. On the contrast, at a very short time interval *B t* << *A* and so,

$$Lt\left(\text{d}M_{t}/\text{d}t\right)=1/A\;t\to 0$$

Therefore, the intercept *A* is reciprocal of initial drug release rate. Finally the Schott kinetic rate constant *k*_2_ is calculated as:

(7) $$k_{2}={\text{Slope}}^{2}/\text{Intercept}$$

**Figure 11.**
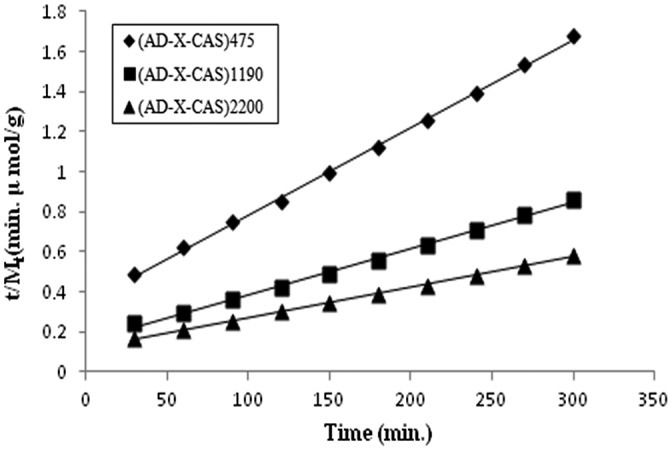
Interpretation of dynamic drug (GS) release data Schott kinetic model.

The *t*/*M*_*t*_ vs. ‘t’ plots for the samples (AD-X-CAS)_475_, (AD-X-CAS)_1190_, and (AD-X-CAS)_2200_ are shown in Figure 11 and the related parameters, evaluated using slopes and intercepts, are given in Table 2.

**Table 2.** Parameters of Schott model.

Sample	*k*_2_ × 10^5^	*M*_∞ (theo)_	*R*_(theo)_	*M*_∞ (exp)_	*R*_(ini)_
(AD-X-CAS)_475_	5.613	227.272	2.899	271.540	2.057
(AD-X-CAS)_1190_	3.419	434.782	6.464	432.769	4.139
(AD-X-CAS)_2200_	1.931	666.666	8.583	584.739	5.965

It can be seen that the regression values for all the three samples are fairly high, thus indicating the suitability of the Schott model. It is also noteworthy that *M*_∞ (theo)_ values are quite close to the *M*_∞ (exp)_ values. In addition, the theoretical and experimental initial release rates i.e. *R*_ini (theo)_ and *R*_ini (exp)_ values are also close to each other. It is also noteworthy that theoretical values are greater than the experimental ones. This may probably be attributable to the fact that in the present study, the cationic drug GS binds to the negatively charged –COO^−^ groups of the casein chains, thus resulting in the lower drug release than expected.

#### 3.4.1. Diffusion coefficients

Most of the diffusion processes are best interpreted by the Fick’s first and second law of diffusion. The initial diffusion coefficient *D*_i_ gives an idea about the drug release from the hydrogel matrix in the initial stage. In order to determine *D*_i_, the following equation was employed [31]:

(8) $$F=\frac{M_{t}}{M_{\infty }}=4{\left[\frac{D_{\text{i}}}{\pi \delta^{2}}t\right]}^{2}$$

where *F* is the fractional release and *D*_i_ is the initial diffusion coefficient. The above equation can be re-arranged as:

The slope of linear plot between *F* and *t* ^1/2^ was used to calculate *D*_i_ as:

(9) $$D_{\text{i}}={\text{Slope}}^{2}\cdot \delta^{2}\pi /16$$

The initial 60% of the drug release data were used to draw linear plots between ln *F* and *t* ^1/2^ (data not shown). The slopes of the linear plots were used to calculate initial diffusion coefficients *D*_i_.

In order to calculate the average diffusion coefficient *D*_ave_, *F* = 0.5 and *t* = *t*_1/2_ were substituted in Equation (8), presuming that 50% of the total release could enable us to evaluate *D*_ave_. The above substitutions yielded following expression:

(10) $$D_{\text{ave}}=0.049\delta^{2}/t_{1/2}$$

To calculate *D*_ave_, the dynamic drug release data were used to evaluate the time required for attainment of 50% of the total release from the various film samples.

**Figure 12.**
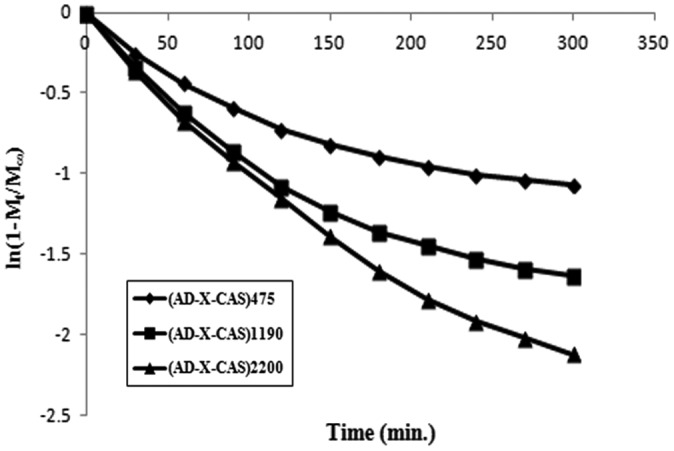
Bi-phasic curve obtained between ln (1 − *M*_*t*_/*M*_∞_) and ‘t’ for various samples.

Finally, the late time diffusion coefficient *D*_L_ was determined using later 60% of the total kinetic release data for various samples. The ln (1 − *M*_*t*_/*M*_∞_) values were plotted against ‘t’ for various samples. The linear plots obtained between ln (1 − *M*_*t*_/*M*_∞_) and ‘t’ were bi-phasic (see Figure 12).

The slopes, obtained for the later part of the linear plots, were used to calculate *D*_L_ using the following expression [31]:

(11) $$D_{\text{L}}=-\left(\text{Slope}\times \delta^{2}\right)/\pi^{2}$$

The various types of diffusion coefficients, discussed above, are given in Table 3. It can be seen that all of the diffusion coefficients are of the order of 10^−7^ as has also been reported in a number of studies.[32] However, in some reports, diffusion coefficients with relatively higher order of order of 10^−4^ have also been reported. For example, Singh and Chauhan [33] has reported release of model drug 5-fluorouracyl from poly(hydroxyethyl methacrylate-co-acrylic acid) hydrogels. The various diffusion coefficients, namely initial, average and late time, were found in the range of 10^−4^ cm^2^ min^−1^. It is also noteworthy that values for initial diffusion coefficients *D*_i_ are much greater than the late time diffusion coefficients *D*_L_ which may probably be attributable to the fact that initially the concentration gradient between the drug on the surface of the film and release medium is very high, thus causing a faster release in the initial phase. However, after an appreciable quantity of drug has released (i.e. fractional release of 0.6), the concentration gradient within the film (along the thickness) is quite low and therefore a slower release is observed.

**Table 3.** The various types of diffusion coefficients.

Sample	*D*_i_ × 10^6^	*D*_(ave)_ × 10^6^	*D*_L_ × 10^6^
(AD-X-CAS)_475_	27.14	71.14	10.33
(AD-X-CAS)_1190_	38.68	70.04	15.52
(AD-X-CAS)_2200_	51.75	79.24	32.41

### 3.5. BSA adsorption onto film

**Figure 13.**
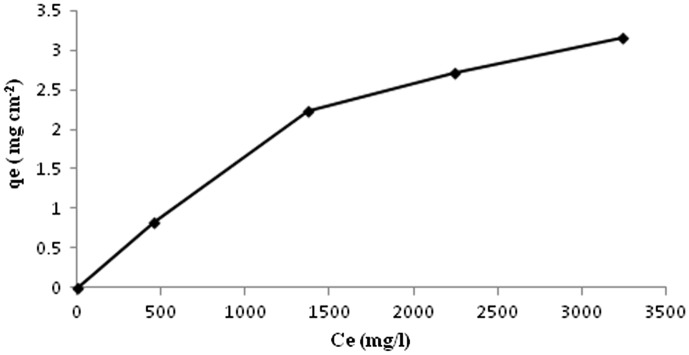
The equilibrium adsorption data for BSA, obtained using a representative film sample (AD-X-CAS)_64_.

The BSA adsorption study determines the extent of BSA sorbed on the film, which has a correlation to the blood compatibility of the film. When a biomaterial first comes into contact with blood, plasma proteins adsorb at the polymer surface. The adsorbed proteins cause platelets to adhere to the surface, which causes a further cascade of platelet activation, aggregation, and fibrin polymerization. Platelets do not readily adhere to serum albumin, so the adsorption of albumin on a material surface will help to prevent thrombus formation. Albumin also diffuses to the surface of a material faster than fibrinogen, and so the ability of albumin to adsorb to a material surface and not be dislodged by fibrinogen molecules is of importance.[34] Kaelble and Moacanin [35] have shown through surface energy analysis that the adsorption and retention of a plasma protein film allows for greater blood compatibility for a material. Therefore, the adsorption capacity of BSA on the fibroin film will be an indicator of the thrombotic potential and biocompatibility of the film. In order to eliminate the effect of varying thickness of the same film sample on the adsorption capacity of BSA, we calculated the adsorption in the terms of g per centimeter square (g cm^−2^) surface area of the film. For proteins interacting with a biomaterial surface, the affinity between the protein and the surface can be represented by an adsorption constant. The equilibrium adsorption data for BSA, obtained using a representative film sample (AD-X-CAS)_64_, is shown in Figure 13. It can be noticed that BSA uptake increases and finally attains optimum value of almost 3.1 mg cm^−2^. The quantity of BSA adsorbed appears to be fairly higher than those reported by others. For example, Kenawy et al. [36], reported that poly(vinyl alcohol)/hydroxyethyl starch blend membrane adsorbed 1.125 mg cm^−2^ of BSA. The same group of workers have also reported BSA adsorption of 1.8 mg cm^−2^ onto poly(vinyl alcohol)/SA blend.[37] A plausible explanation for a fairly higher degree of BSA adsorption, in the present work may be attributed to the various interaction forces between protein molecules and the membrane surface, such as weak bonding (van der Waal interactions), ionic bonding and hydrogen bonding. It is noteworthy that both, the adsorbent components, i.e. casein molecules and dialdehyde alginate contain polar groups. Similarly adsorbate BSA also have a number of polar functionalities. Therefore strong interactions between the film constituents and the protein BSA are most probable. The adsorption data was applied on the two basic isotherm models, namely Langmuir and the Freundlich isotherm.[38] The Langmuir model may be given as:

(12) $$q_{\text{e}}=\frac{Q_{\text{o}}}{1+}\frac{K_{\text{L}}}{K_{\text{L}}}\frac{C_{\text{e}}}{C_{\text{e}}}$$

where *C*_e_ = Equilibrium concentration of drug solution (mg/L); *Q*_o_ = Maximum monolayer sorption capacity (mg/m^2^); *K*_L_ = Langmuir isotherm constant (L mg^−1^).

The BSA adsorbed at equilibrium (i.e. *q*_e_) may be calculated using the following expression:

(13) $$q_{\text{e}}=\frac{C_{0}-C_{\text{e}}}{A}\times V$$

where *C*_0_ = Initial concentration of BSA solution (mg/L); *A* = Area of the test film sample (cm^2^); *V* = Volume of protein solution taken for adsorption study (L).

In order to determine *q*_e_ in terms of mg/g film the above equation was transformed in to:

(14) $$q_{\text{e}}=\frac{C_{0}-C_{\text{e}}}{w}\times V$$

where *w* = weight of the film in g.

The linearized form of the above equation is:

(15) $$\frac{C_{\text{e}}}{q_{\text{e}}}=\frac{C_{\text{e}}}{Q_{\text{o}}}+\frac{1}{Q_{{\text{o}K}_{\text{L}}}}$$

**Figure 14.**
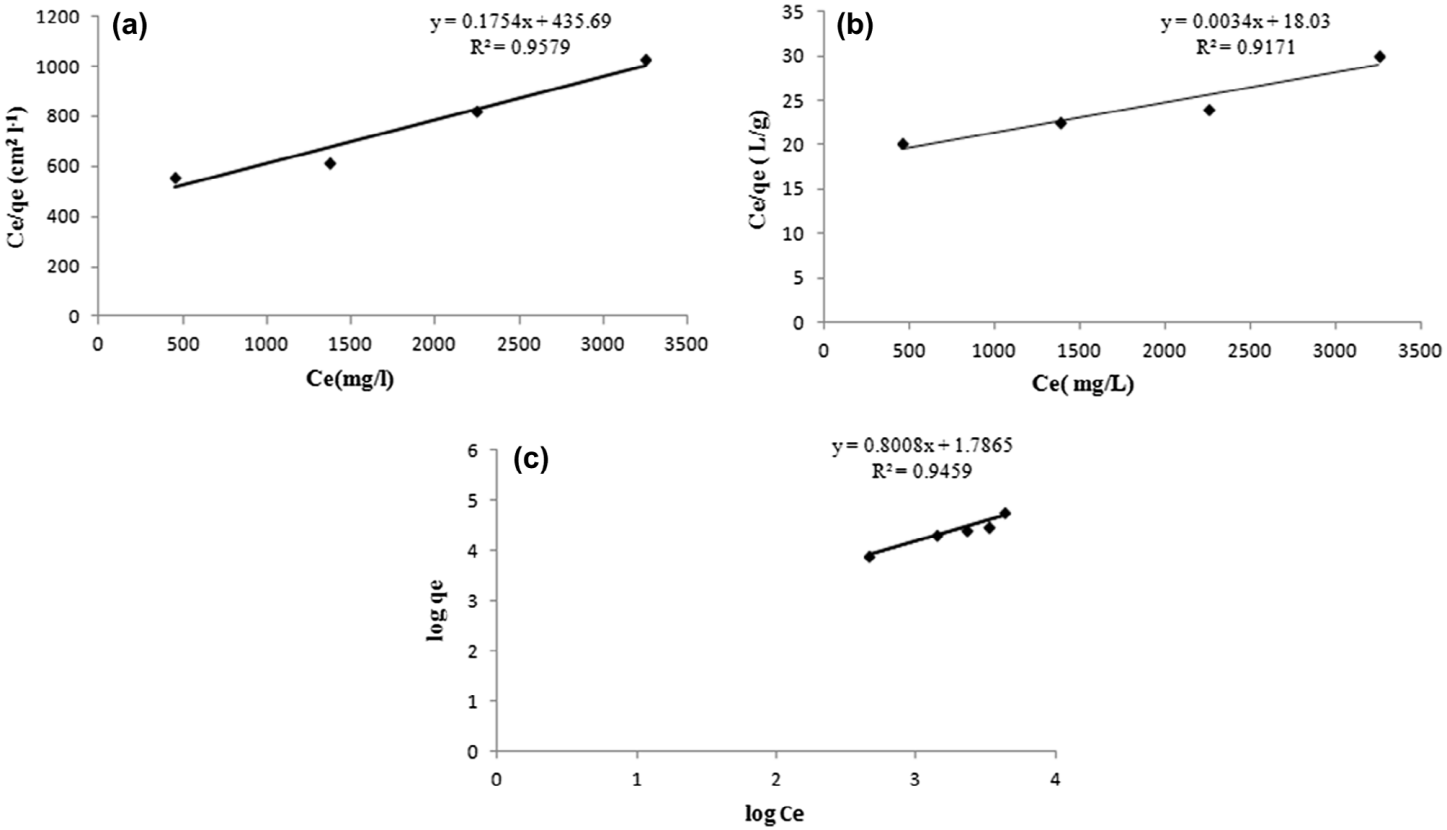
The linearized forms of the Langmuir model for adsorption capacity expressed as (a) mg/cm^2^ and (b) mg/g; (c) slope of Freundlich plot showing value for (1/*n*) equal to 0.88, indicating a normal adsorption.

Therefore, a linear plot between *C*_e_/*q*_e_ and *C*_e_ enables to evaluate the related parameters *Q*_o_ and *K*_L_. The Freundlich isotherm is given as:

(16) $$\text{Log}\;q_{\text{e}}=\text{log}\;K_{\text{F}}+\left(1/n\right)\text{log}\;C_{\text{e}}$$

where *K*_F_ (mg/g (l/mg)^1/*n*^) is a Freundlich constant related to sorption capacity and ‘n’ refers to sorption intensity. The constant *K*_F_ indicates adsorption capacity, while 1/*n* is a function of the strength of adsorption in the adsorption process. The linearized forms of the Langmuir model were obtained by plotting *C*_e_/*q*_e_ vs. *C*_e_ as shown in Figure 14(a) and (b) for adsorption capacity expressed as mg/cm^2^ and mg/g respectively.

The regressions for the two respective plots were 0.9579 and 0.9171 respectively, thus indicating a better suitability of the model with *q*_e_ expressed as mg/cm^2^. The maximum sorption capacity, as calculated using the slope of the Langmuir plot, was 5.701 mg per cm^2^ area and 294.11 mg/g of the film respectively. Since, the film with 1 cm^2^ area possessed a mass of 0.357 g, the BSA adsorbed by 0.357 g of the film (with area as 1 cm^2^) was 10. 29 mg. In other words, more BSA was adsorbed when calculation is made on the basis of mass of the film. This clearly indicates that during the sorption process, BSA molecules, apart from getting adsorbed on the surface, must have diffused in to the film matrix also. Finally, using the slope of the Freundlich plot, given in Figure 14(c), the value of (1/*n*) was found to be 0.88, thus indicating a normal adsorption.[39]

### 3.6. Oxygen permeability

**Figure 15.**
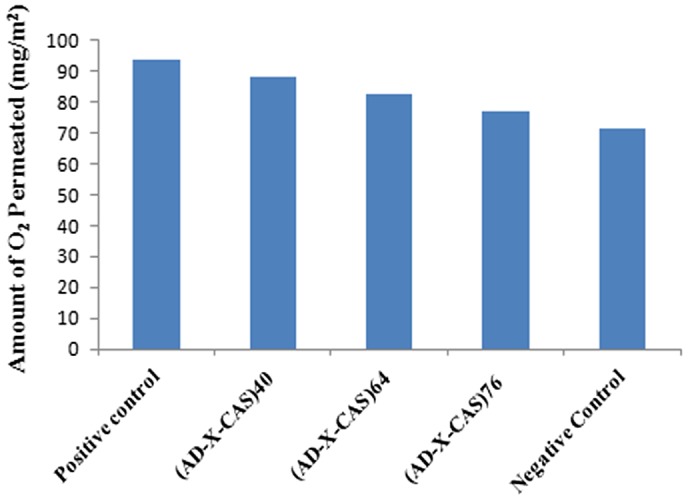
Bar diagram showing O_2_ permeated through different film samples.

Oxygen plays a very important part in the wound healing process, and as such an important property of a wound dressing is adequate OP to the site of the wound. The major role of oxygen at the site of a wound is to control the growth of anaerobic bacteria and reduce the risk of infection, as well as decreasing tissue necrosis. Many of the cells and processes in the wound healing process require oxygen, and there are some evidences that suggest increased exposure to oxygen at the wound site helps to oxygenate wound tissue and aids healing.[40] The results of O_2_ permeability are shown in Figure 15. It can be seen that as the degree of crosslinking increases, there is slight decrease in the amount of oxygen permeated. This is attributable to the fact that with the increase in the crosslinker concentration within the film, the mesh size becomes more and more narrow, thus putting hindrance in the flow of oxygen through the film. Although, decrease in the O_2_ permeated is quite marginal.

### 3.7. Antibacterial test

**Figure 16.**
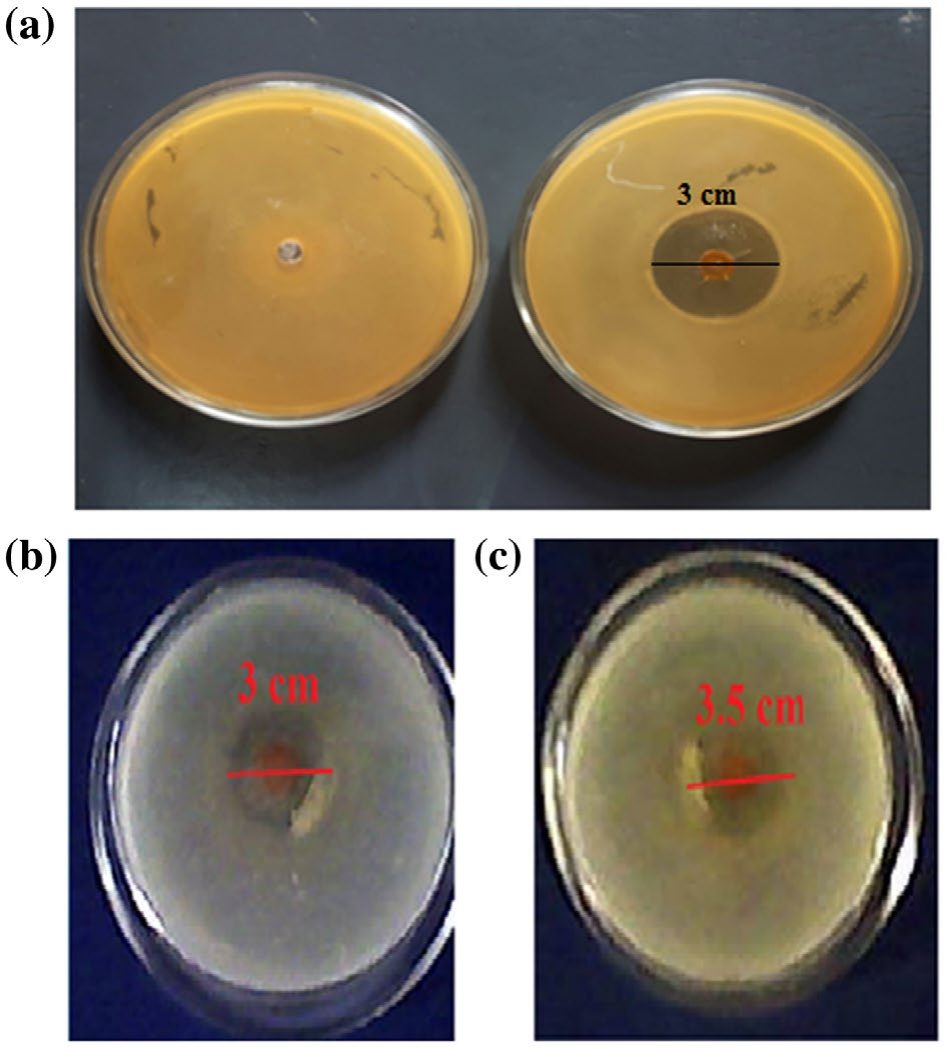
(a) Antibacterial activity of plain (AD-X-CAS) and drug loaded (AD-X-CAS)_1190_ films against model bacteria *E. coli* and anti-fungal activity of the drug-loaded sample (AD-X-CAS)_1190_ against (b) *C. albicans*, and (c) *C. parapsilosis*.

The results of antibacterial experiments against model bacteria *E. coli* are shown in the Figure 16(a). It can be seen that the Petri plate, containing the plain film, shows a dense population of bacterial colonies whereas the Petri plates, supplemented with the hydrogel films (AD-X-CAS)_1190_ shows a ‘zone of inhibition’ with a diameter of 3 cm. This indicates that drug GS-loaded film shows fair antibacterial activity.

### 3.8. Anti-fungal test

The anti-fungal activity of the drug-loaded sample (AD-X-CAS)_1190_ was tested by observing their antifungal activity against *C. albicans* and *C. parapsilosis*. Figure 16(b) and (c) show the typical antifungal test results obtained for the films by the disc method. The sample (AD-X-CAS)_1190_ demonstrated inhibition zones of nearly 3 and 3.5 cm for *C. albicans*, and *C. parapsilosis* respectively *.*However, no such zones were obtained for the plain sample (data not shown).

### 3.9. Wound healing study

**Figure 17.**
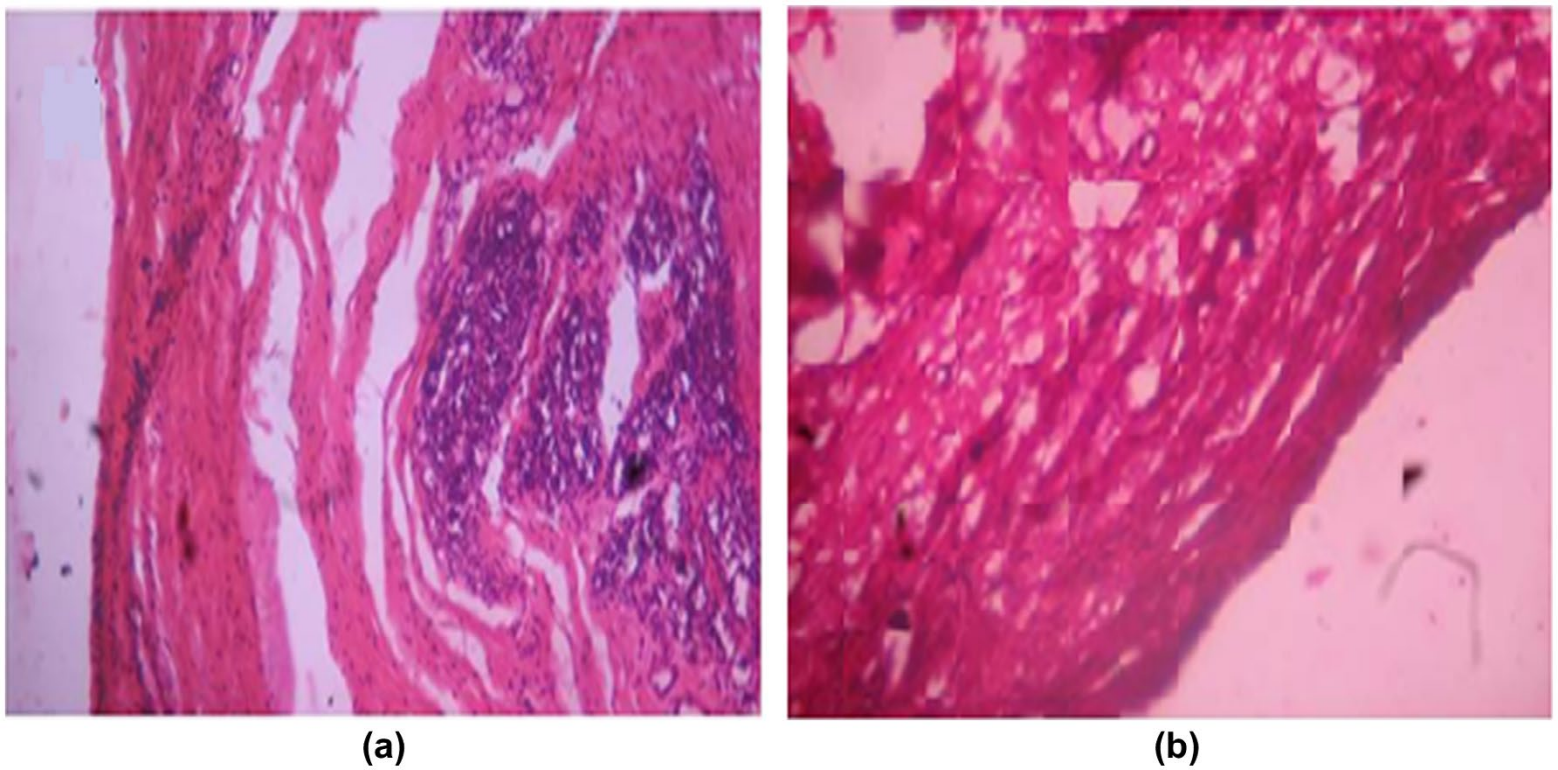
Wound healing activity of (a) plain (AD-X-CAS) film (b) GS loaded (DA-X-CAS)_1190_ film.

Wounds treated with plain film patch showed poor indication of dermal healing as compared to Group II animals. Histologically, wounds treated with blank film revealed less collagen content and more inflammatory collections (see Figure 17(a)). On the other hand wound treated with (DA-X-CAS)_1190_ patch revealed remarkably less scar at the closure of wounds. There was increase in collagen fibers with negligible inflammatory collection (Figure 17(b)).

## 4. Conclusion

This study demonstrates a diffusion controlled release of antibacterial drug gentamicin from the dialdehyde alginate-crosslinked gelatin films, with release exponents between 0.43 and 0.45. The dynamic release is best interpreted by the Schott kinetic model. The equilibrium adsorption of therapeutic protein BSA was best fitted to Langmuir isotherm model, with the maximum sorption capacity of 5.701 mg per cm^2^ area of the film and 294.11 mg per g weight of the film respectively. The O_2_ permeability showed a slight decrease with the degree of crosslinking of the films. Finally, in the animal study on Albino wistar, wound treated with gentamicin loaded patch revealed remarkably less scar at the closure of wounds, with increase in collagen fibers and negligible inflammatory collection. These films have great potential for wound healing.

## Disclosure statement

No potential conflict of interest was reported by the authors.
